# A Bacterial Quorum Sensing Regulated Protease Inhibits Host Immune Responses by Cleaving Death Domains of Innate Immune Adaptors

**DOI:** 10.1002/advs.202304891

**Published:** 2023-10-23

**Authors:** Xiangke Duan, Zhao Zhi Boo, Song Lin Chua, Kelvin Han Chung Chong, Ziqi Long, Renliang Yang, Yachun Zhou, Baptiste Janela, Sanjay Haresh Chotirmall, Florent Ginhoux, Qinghua Hu, Bin Wu, Liang Yang

**Affiliations:** ^1^ Shenzhen Third People's Hospital The Second Affiliated Hospital of Southern University of Science and Technology National Clinical Research Center for Infectious Disease Shenzhen 518112 P. R. China; ^2^ School of Medicine Southern University of Science and Technology Shenzhen Guangdong 518055 P. R. China; ^3^ Shenzhen Center for Disease, Control and Prevention Shenzhen 518055 P.R. China; ^4^ School of Biological Sciences Nanyang Technological University Singapore 637551 Singapore; ^5^ NTU Institute of Structural Biology Nanyang Technological University Singapore 636921 Singapore; ^6^ Department of Applied Biology and Chemical Technology The Hong Kong Polytechnic University Hong Kong SAR 999077 P. R. China; ^7^ Skin Research Institute of Singapore Singapore 308232 Singapore; ^8^ Singapore Immunology Network Agency for Science, Technology and Research (A*STAR) 8A Biomedical Grove, Immunos Singapore 138648 Singapore; ^9^ Lee Kong Chian School of Medicine Nanyang Technological University Singapore 639798 Singapore

**Keywords:** host‐microbe interaction, innate immune adaptor MyD88, protease, *Pseudomonas aeruginosa*, quorum sensing

## Abstract

Innate immune adaptor proteins are critical components of the innate immune system that propagate pro‐inflammatory responses from their upstream receptors, and lead to pathogen clearance from the host. Bacterial pathogens have developed strategies to survive inside host cells without triggering the innate immune surveillance in ways that are still not fully understood. Here, it is reported that *Pseudomonas aeruginosa* induces its quorum sensing mechanism after macrophage engulfment. Further investigation of its secretome identified a quorum sensing regulated product, LasB, is responsible for innate immune suppression depending on the MyD88‐mediated signaling. Moreover, it is showed that this specific type of pathogen‐mediated innate immune suppression is due to the enzymatic digestion of the death domains of the innate immune adaptors, mainly MyD88, and attributed to LasB's large substrate binding groove. Lastly, it is demonstrated that the secretion of LasB from *P. aeruginosa* directly contributed to MyD88 degradation within macrophages. Hence, it is discovered an example of bacterial quorum sensing‐regulated cellular innate immune suppression by direct cleavage of immune adaptors.

## Graphical abstract

MyD88 is a crucial adapter protein in the innate immune response that plays a significant role in the recognition and response to microbial infections. The *Pseudomonas aeruginosa* quorum sensing regulated effector LasB can suppress the innate immune response through direct cleavage of the MyD88 immune adaptor and facilitate intracellular survival within host cells.

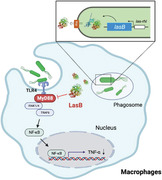

## 1 Introduction

The spread of drug‐resistant bacteria globally is associated with increased morbidity, mortality, healthcare costs and boosts the development of alternative strategies for combating bacterial infections. One promising approach for controlling bacterial infections is the innate immunomodulation therapy which precisely activates the disease‐specific protective antimicrobial defense. To establish successful infection and prevent the full activation of host innate immune responses, pathogens shed toxic factors and enzymes that cleave and inhibit the innate immune signaling mediators, thereby suppressing overall immune responses.^[^ ^1^ ^]^ For example, *P. aeruginosa* secrets various immune toxic factors modulating the innate immune system.^[^ ^2^ ^]^ This scenario is further exacerbated when host immunity is suppressed, perpetuating the establishment and progression of primary and secondary infections.^[^ ^3^ ^]^ Viral and bacterial infections are sensed by diverse innate immune receptors such as Toll‐like receptors (TLR) and NOD‐like receptors (NLR).^[^ ^4^ ^]^ Being triggered by their ligands, these receptors become activated and initiate signaling cascades that are propagated by their corresponding downstream adaptors.^[^ ^5^ ^]^ Most of the receptors and the adaptor proteins contain death domains, which act through homotypic and heterotypic protein‐protein interactions that induce Death Domain oligomerization.^[^ ^6^ ^]^ Myeloid differentiation primary response 88 (MyD88) is the adaptor protein downstream of most of the TLRs,^[^ ^7^ ^]^ except TLR3, while apoptosis‐associated speck‐like protein containing a CARD (ASC, also known as PYCARD) is the downstream adaptor protein for many NLRs.^[^ ^8^ ^]^ Both of these two adaptor proteins are known to form innate immune signalosomes, named myddosome and inflammasome, once stimulated by upstream signals. Myddosomes and inflammasomes further recruit and activate downstream effectors, such as IRAK4 and pro‐caspase 1, through Death Domain mediated oligomerization.^[^ ^6a,c^ ^]^ The perpetual signaling of these signalosomes allows a massive amplification of the initial signal, leading to a rapid and strong innate immune response. There are hardly any effective ways to break down the signaling‐active signalosomes composed of oligomerized innate immune adaptor proteins.^[^ ^9^ ^]^ Structure analysis revealed that the TIR domains of MAL and MyD88 form homodimers and heterodimers, which are stabilized by conserved hydrophobic residues.^[^ ^9b^ ^]^ The TIR domain of MAL (MAL^TIR^) promotes unidirectional assembly of MyD88 TIR.^[^ ^9a^ ^]^ Pathological point mutations of MyD88 result in both loss and gain of function, and L252P mutants form stable oligomers that would be therapeutically attractive targets.^[^ ^10^ ^]^ However, if the oligomerization process of these key signalosomes were somehow disrupted, cellular innate immune signaling collapsed.^[^ ^11^ ^]^ This provides an excellent opportunity to harness the innate immune response.

Existing knowledge of pathogenic immunosuppression of PRR‐adaptor signal transduction are primarily derived from viral infection studies. The 3C is a viral cysteine protease that disrupts host cell function by cleaving host cell factors. Indeed, the 3C of both picornavirus and encephalomyocarditis virus is involved in the disruption of innate immune signaling through RIG‐I cleavage.^[^ ^12^ ^]^ Another example is the Hepatitis C virus (HCV) NS3/4A protease,^[^ ^13^ ^]^ which is a heterodimer complex consisting of a serine protease (NS3) and an activating co‐factor (NS4A).^[^ ^14^ ^]^ It prevents the induction of type‐1 interferons by inhibiting Interferon Regulatory Factor‐3 signaling and cleaving the upstream adaptor protein MAVS.^[^ ^13^, ^15^ ^]^ By disrupting the innate immune signaling at the adaptor protein level, it will allow persistent infection and expose the host cell to secondary infection by viruses that would otherwise be detected and removed.

Similar to viruses, many bacterial species could survive intracellularly in their host.^[^ ^16^ ^]^ The opportunistic pathogen *P. aeruginosa* secretes several quorum sensing‐regulated proteases, such as alkaline protease (AprA) and elastase B (LasB), to degrade mammalian proteins and cause infections such as pneumonia and keratitis.^[^ ^17^ ^]^ The effectors of type 3 secretion system (T3SS), including ExoY, ExoS and ExoT can inhibit the inflammasome activation and phagocytosis of macrophage by delaying the activation of NF‐κB and caspase‐1, which prevent the clearance of *P. aeruginosa* form the host.^[^ ^18^ ^]^ The virulence factor MgtC contributes to the intracellular survival of *P. aeruginosa* within macrophages via the activation of T3SS.^[^ ^19^ ^]^ The effector of T1SS TesG inhibits the MYPT‐1 and JNK signaling by targeting the eukaryotic small GTPase RhoA, therefore suppress the inflammatory response of host.^[^ ^2b^ ^]^ It also produces a plethora of cell‐associated or extracellular virulence factors, such as exotoxins, rhamnolipids, exopolysaccharides and lipopolysaccharides (LPS),^[^ ^20^ ^]^ which could impair immune functions ranging from blood coagulation to degradation of the extracellular matrix.^[^ ^21^ ^]^ Hence, there is increasing evidence indicating that bacterial intracellular survival is dependent on innate immune suppression, which is believed to be a target for developing anti‐virulence drugs against drug resistant pathogens.^[^ ^22^ ^]^

In this study, we observed that *P. aeruginosa* is able to survive inside macrophages after engulfment and induce quorum sensing mechanisms via a metabolic labeling proteomics approach. We next took a reconstitution approach to isolate the bacterial factors that are capable of quenching host immune signaling at the receptor‐adaptor stage. After screening the bacterial secretomes, we identified that the *P. aeruginosa* quorum sensing‐regulated product LasB could digest and prevent the oligomerization of multiple innate immune adaptors, thus silencing the immune response during their opportunistic intracellular infection. We propose that LasB and other related bacterial proteases play important roles in establishing the chronic cellular infection. In addition, our investigation also shed light on the mechanisms of how bacterial proteases can degrade and remove the Death Domain protein oligomers in host cells, thereby helping bacteria to evade the innate immune response.

## 2 Results

### 2.1 *P. aeruginosa* Induces Quorum Sensing Mechanisms After Macrophage Engulfment

**Figure 1:**
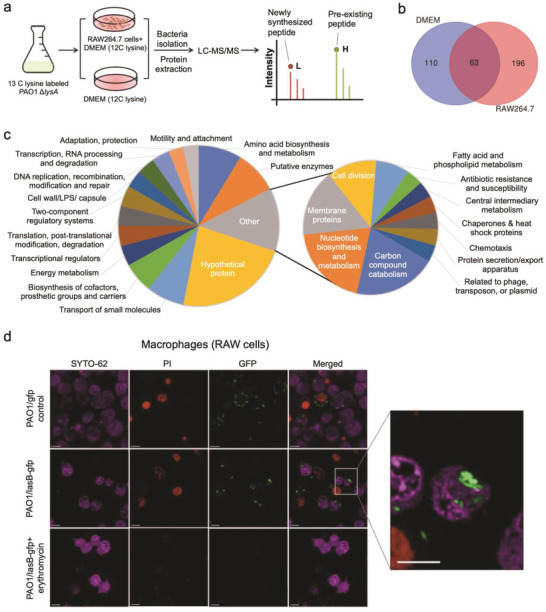
*P. aeruginosa* induces quorum sensing mechanisms after macrophage engulfment. a) Experimental design for *P. aeruginosa* protein turnover measurements during infection. b) The Venn diagram illustrating the newly synthesized proteins in DMEM group and RAW264.7 cells group. c) Function classification of newly synthesized proteins in intracellular bacteria. d) Confocal microscopy image of PAO1 infection of live RAW264.7 macrophages. *P. aeruginosa* PAO1/pLasB‐gfp expressing LasB‐GFP was detected in live RAW264.7 macrophages. Upon erythromycin treatment, LasB‐GFP was not observed. Experiments were performed in triplicate, and a representative image for each condition was shown. Enlarged view of LasB‐GFP tagged PAO1 showed that LasB was released into cytoplasm of the host cell. Scale Bars, 10 µm.

To identify the newly synthesized proteins by *P. aeruginosa* after engulfment by host immune cells, we employed the pulsed‐SILAC proteomics approach to label the bacterial proteome with L‐lysine‐^13^C_6_^15^N_2_ (Lys8) and L‐lysine (Lys0) before and after entering host cells, respectively. The PAO1△*lysA* mutant which failed to synthesize lysine was cultured in ABTG medium^[^ ^23^ ^]^ supplemented with stable isotope‐containing amino acid L‐lysine‐^13^C_6_^15^N_2_ (Lys8). The Lys8 labeled bacteria were then harvested and used to infect the RAW264.7 cells which were cultured in DMEM containing regular L‐lysine (Lys0). After 4 h infection, RAW264.7 cells were lysed, and the intracellular bacteria were harvested and analyzed by LC‐MS/MS. For the control DMEM group, labeled bacteria were diluted in pure DMEM medium (**Figure** **1**a). By using this pulsed‐labelling approach, the newly synthesized proteins can be selectively tagged with Lys0 during the infection. Remarkably, up to 196 proteins have been identified as intracellular specific newly synthesized proteins (Figure 1b; Table S1, Supporting Information). Those proteins were functionally grouped (Figure 1c,). We noticed that a series of virulence related proteins were synthesized during the 4 h infection intracellularly, including the well‐known quorum sensing regulator LasR (Table S1, Supporting Information).

To validate the activation of the *las* quorum sensing (QS) mechanisms of *P. aeruginosa* after macrophage engulfment, we next investigated whether the *las* quorum sensing‐reporting system is indeed expressed during intracellular infection. RAW264.7 cells infected with *P. aeruginosa* containing a widely used translational report fusion (p*lasB*‐*gfp*)^[^ ^24^ ^]^ showed GFP signals within the cytoplasm of RAW264.7 cells (Figure 1d), indicating the upregulated transcription of *lasB* gene and production of LasB‐GFP. Erythromycin has been clinically used for long periods for treating *P. aeruginosa* infections despite the lack of antibacterial activity, which was more recently shown can reduce LasB production by quorum sensing inhibition.^[^ ^25^ ^]^ What's more, macrolide antibiotics such as erythromycin have exceptionally high levels of accumulation and retention in cells, especially phagocytes.^[^ ^26^ ^]^ Conversely, infected cells treated with erythromycin did not show any GFP signal. Loss of GFP signal in the presence of erythromycin indicates that the GFP signal observed is indeed LasB‐GFP (Figure 1b). This result indicates that *P. aeruginosa* is actively expressing its quorum sensing mechanisms after macrophage engulfment, which might lead to secretion of abundant of quorum sensing regulated virulence products to modulate the host cell functions from inside.

### 2.2 *P. Aeruginosa* Secretion Suppresses Innate Immune Signaling by Quenching Activation of Cytosolic Innate Immune Adaptors

**Figure 2:**
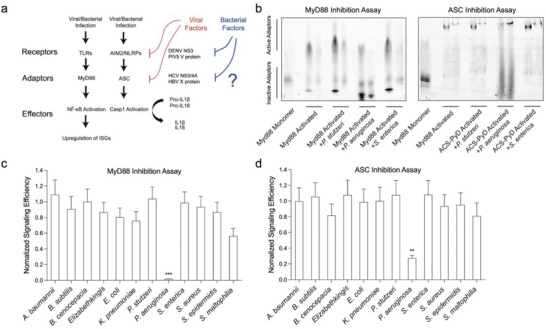
*P. aeruginosa* secretion suppresses innate immune signaling by quenching the activation of cytosolic innate immune adaptors. a) Classical Innate Immune Signaling sensing intracellular pathogens. Various viruses developed mechanisms to quench the signaling by disrupting the activation and oligomerization of their corresponding receptors and adaptors, highlighted in red. Such molecular mechanisms in innate immune suppression by opportunistic and latent intracellular bacterial infection is less understood, highlighted in blue. b) Representative images of the innate immune inhibition assays used in our in vitro screens. MyD88 Pyrin domain (MyD88) and ASC Pyrin domain (ASC‐PyD) were tagged with a fluorescently labelled SNAP tag. The activation state of these recombinant adaptor proteins were accessed by native blue page EMSA. Without addition of bacterial secretions, the monomeric adaptor protein at resting state could be activated by mixing with their corresponding activated signaling “seeds”, and oligomerized and exhibited retarded migration rate on native gel. Addition of bacterial secretions containing anti‐inflammatory suppressors could prevent the oligomerization process. Two concentrations (0.7 µM and 0.25 µM) of monomer adaptor protein were mixed with pre‐activated “seeds” to monitor the innate immune signaling capacity. Bacterial secretions affecting ASC‐PyD signaling was also assessed using the native gel EMSA. c) *P. aeruginosa* secretions strongly inhibit MyD88 signaling. Gel images band intensities were quantified by ImageJ. Raw data attached in Figure S2 (Supporting Information). Mean ± standard error is from three independent experiments. ***p< 0.001, One‐way ANOVA. d) *P. aeruginosa* secretions strongly inhibit ASC‐PyD signaling. Raw data attached in Figure S3 (Supporting Information). Mean ± standard error is from three independent experiments. **p< 0.01, One‐way ANOVA.

The QS system controls the expression of a variety of virulence factors, including rhamnolipids, lectin, toxins, proteases, such as elastase B (LasB), protease IV (PIV), and elastase A (LasA). Alkaline protease (AprA) contributes to *P. aeruginosa* interfere the host immune response by degrading cytokines (INF‐γ, TNF‐α and IL‐6) and complement proteins (C1q, C2 and C3).^[^ ^27^ ^]^ LasB can also modulates the host innate and adaptive immune defense via degrading INF‐γ, TNF‐α, IL‐2 and monocyte chemotactic protein‐1 (MCP‐1).^[^ ^28^ ^]^ These factors contribute to the ability of *P. aeruginosa* to cause tissue damage, evade the host immune response, and establish infections.^[^ ^29^ ^]^ MyD88 is a crucial adapter protein in the innate immune response that plays a significant role in the recognition and response to *P. aeruginosa* infection.^[^ ^30^ ^]^ In the context of macrophages, which are immune cells that play a central role in detecting and eliminating infections, MyD88 is involved in mediating the response to *P. aeruginosa* through Toll‐like receptors (TLRs). Upon recognition of *P. aeruginosa* by TLR4, the TLR4 receptor complex recruits MyD88. MyD88 acts as an adapter protein, facilitating the recruitment and activation of other proteins, including IRAK (Interleukin‐1 Receptor‐Associated Kinase) family members. The activation of IRAK proteins triggers a series of phosphorylation events and protein‐protein interactions, ultimately leading to the activation of transcription factors such as NF‐κB (Nuclear Factor‐Kappa B) (**Figure** **2**a). These transcription factors drive the expression of genes involved in the inflammatory response, such as proinflammatory cytokines and chemokines.^[^ ^31^ ^]^ To explore the potential innate immune modulating effects of bacterial secreted proteases via degrading the immune adapter proteins, a modified protocol described in Wu, et al.^[^ ^32^ ^]^ was used to controllably recapitulate in vivo signal propagation (Figure S1, Supporting Information). We labelled the seed MyD88‐DD with BG‐647 and IRAK2‐DD with BG‐488, and visualized under two different channels. By examining the slowed migration of IRAK2, we could infer which MyD88‐DD is active. To mimic innate immune adaptor activation, pre‐activated MyD88 Death domain (DD) complexes were used to activate and recruit resting state MyD88 DD monomers.^[^ ^7b^ ^]^ This assay platform was screened against secretions from *P. aeruginosa* and other eleven different species of bacteria. We found that the secretion from *P. aeruginosa* inhibited MyD88 DD and ASC oligomerization in our screen (Figure 2b). Further experiments suggested that the signal inhibition is through the cleavage of the death domains of the immune adaptors (Figure 2c,d), since SDS‐PAGE analysis of the inhibited MyD88 and ACS showed a reduction in molecular size (Figure S2 and S3, Supporting Information).

### 2.3 Biochemical Characterization and Purification of Innate Immune Suppressor from *P. Aeruginosa* Secretion

**Figure 3:**
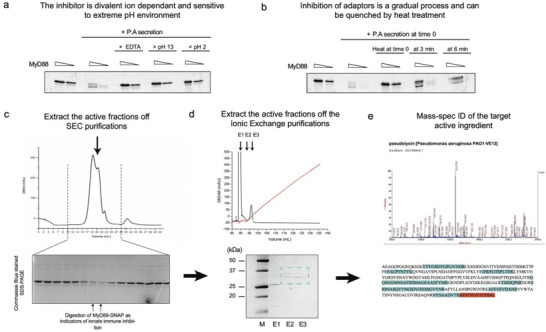
Biochemical characterization and purification of innate immune suppressor from *P. aeruginosa* secretion. a) In vitro assay characterizing biochemical behavior of toxin which inhibited MyD88 activation. *P. aeruginosa* secretions were incubated with purified activated MyD88 in different chemical conditions. MyD88 was titrated at 0.7 and 0.25 µM. Upon exposure to 10 mM EDTA or low and high pH, the digestive activity of *P. aeruginosa* secretion was inhibited. b) In vitro assay characterizing digestive behavior of toxin inhibiting MyD88 activation. *P. aeruginosa* secretions were incubated with purified activated MyD88 and then heated at 95 °C for 3 or 6 mins to quench the reaction. When MyD88 DD SNAP was incubated with pre‐quenched *P. aeruginosa* secretions, no digestion was observed. Quenching the reaction at progressively longer time points showed correspondingly stronger digestion activity. c) Size exclusion chromatography of *P. aeruginosa* secretion. Eluted fractions were incubated with purified activated MyD88 DD and analyzed on SDS PAGE. Black arrows indicated eluted fractions which showed digestive activity towards MyD88. d) Ion exchange chromatography of active fractions obtained from size exclusion chromatography. Analysis of eluted fractions on SDS PAGE showed pure fractions. The band in E3 was sent for mass spectrometry. e) Mass spectrometry of the purified protein (E3) indicated that its identity was LasB. Highlighted amino acid sequences were the peptide fragments identified by mass spectrometry. Identified peptides were colored in blue and red (overlapping region of nearby peptides).

We next aimed to identify and biochemically characterize the *P. aeruginosa* factor responsible for inhibiting MyD88. *P. aeruginosa* secretion mixture was incubated with MyD88 DD complexes, and alterations in buffer conditions (10 mM EDTA, low pH or high pH buffers) were found to have a significant impact on the innate immune suppression activities. SDS‐PAGE analysis showed that these conditions disrupted the cleavage activity that was observed in the assay (**Figure** **3**a), indicating that the inhibitor was ion dependent and sensitive to an extreme pH environment. Furthermore, cleavage of MyD88 DD is time‐dependent and could be quenched by heat treatment at earlier time points of the reaction (Figure 3b). Based on these results, we propose that one or a few of the bacterial metallo‐proteases could potentially cleave the innate immune adaptors.^[^ ^33^ ^]^ We then conducted systematic fractionation to purify this enzyme. By using SEC (superdex 200, Figure 3c) and ionic exchange (heparin, Figure 3d), we successfully isolated two bands that contribute to MyD88 DD degradation activity. Through trypsin assisted mass‐spectrometry analysis, we confirmed that the identity of the enzyme is LasB (Figure 3e), a well‐known *las* quorum sensing‐regulated virulence product of *P. aeruginosa*.^[^ ^34^ ^]^ LasB can degrade a variety of protein substrates of the host, including chemokines, cytokines, surfactants, receptors, and complement components.^[^ ^28^ ^]^

### 2.4 LasB Alone is Responsible for The Innate Immune Suppression Activity of *P. Aeruginosa* Secretion

**Figure 4:**
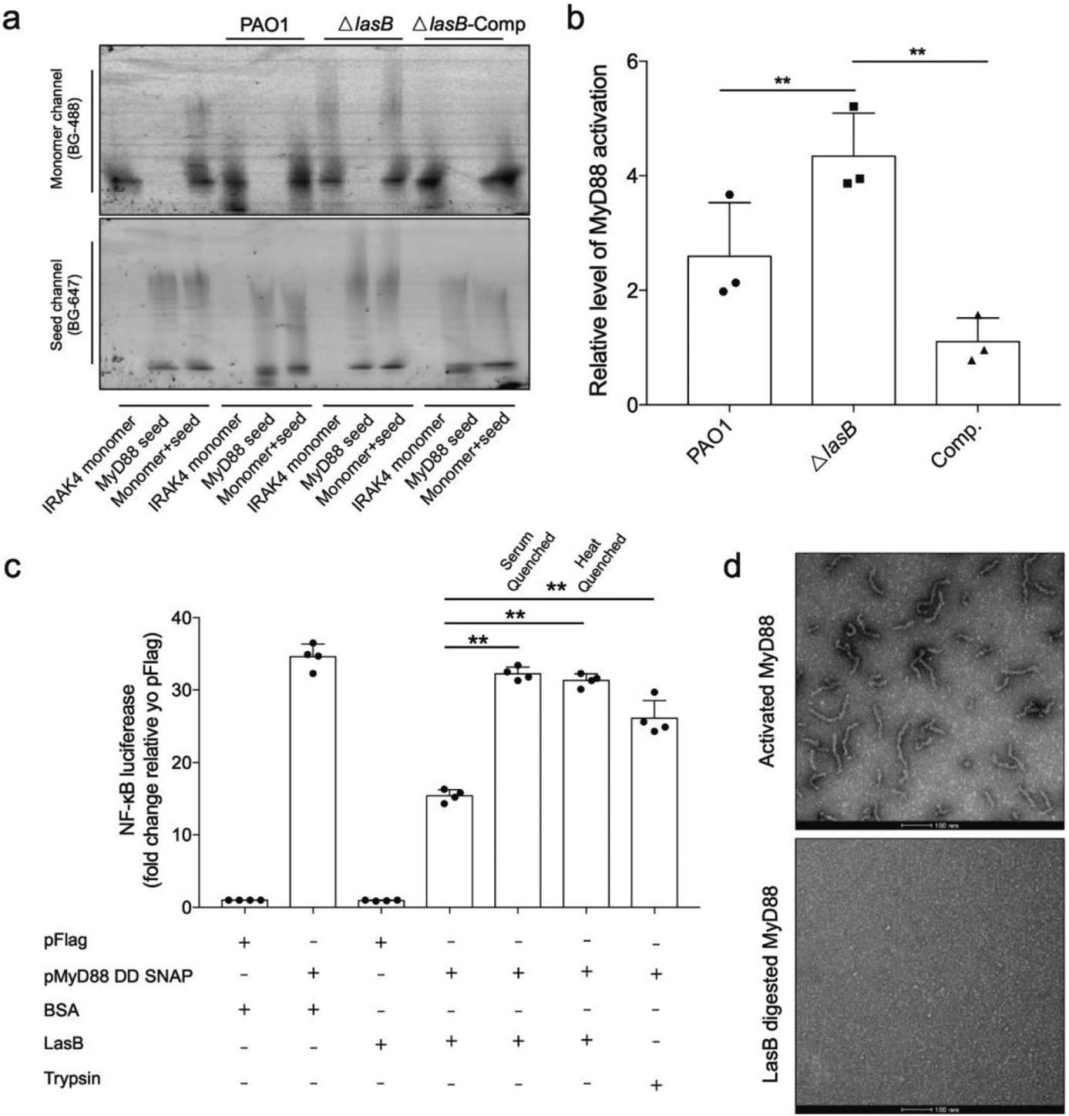
LasB alone is responsible for the innate immune suppression activity of *P. aeruginosa* secretion. a) Native blue page EMSA of SNAP tagged MyD88. Secretions from *P. aeruginosa* strain with LasB knockout (Δ*lasB*) lost the ability to inhibit MyD88 activation. Complementation of the LasB gene (Δ*lasB*/p*_lac_*‐*lasB*) could rescue the phenotype. Raw data attached in Figure S4 (Supporting Information). b) Chart showing quantified values of protein bands from panel A. Bands intensity were quantified using ImageJ and normalized against MyD88 that has not been exposed to *P. aeruginosa* secretions. Reduction in response was not observed when serum‐quenched or heat‐denatured LasB was used. 40 nM of trypsin was also transfected as a control and it did not reduce inflammatory response significantly. Mean ± standard error is from three independent experiments. **p< 0.01, One‐way ANOVA. c) NF‐κB luciferase reporter assay showing inflammatory response of HEK293. HEK293 was stimulated with overexpression of MyD88‐Pyd. Transfection of 40 nM of purified LasB into the cells was sufficient to reduce MyD88‐Pyd‐induced NF‐κB response by almost 2‐fold. Mean ± standard error is from four independent experiments. **p< 0.01, One‐way ANOVA. d) Negative stain electron microscopy images of activated MyD88 DD before and after exposure to 40 nM LasB for 30 min. Experiments were performed in triplicate, and a representative image for each condition was shown. Scale Bars, 100 nm.

*P. aeruginosa* is known to secrete multiple virulence factors.^[^ ^35^ ^]^ To ascertain that secreted LasB plays a predominant role in disrupting MyD88 signaling, knockout strains of *P. aeruginosa* were generated. Secretions of a LasB‐deficient strain (Δ*lasB*) could not disrupt MyD88 signal propagation in vitro, while *lasB* complementation restored the phenotype (**Figure** **4**a).^[^ ^36^ ^]^ To validate if LasB alone was sufficient to stop inflammatory signaling in vivo, purified LasB was electroporated into HEK293T cells. These HEK293T cells with overexpression of MyD88 DD were then assessed by the NF‐κB luciferase reporter assay. The reporter assay showed that 40 nM of LasB was sufficient to reduce the MyD88 DD‐induced inflammatory response by half (Figure 4b). The immune‐suppression activity required direct delivery of LasB into the cells, as serum‐quenched LasB showed little to no immune‐suppression activity. Trypsin control indicated that the proteolytic activity of LasB was specific towards innate immune proteins (Figure 4c). If LasB was pre‐incubated with serum components, or heat treated, such immune‐suppression activity is gone, demonstrating the necessity to deliver well folded LasB directly into the host cell to inhibit innate immune responses (Figure 4c). In addition, we performed negative stain electron microscopy (EM) experiments to confirm that purified pre‐activated MyD88 oligomers was digested into inactive fragments by LasB (Figure 4d). Hence, LasB directly digests the innate immune adapters, thereby suppressing immune response. In addition, MyD88, as compared to other innate immune adaptor proteins, forms relatively smaller and flexible structures (as assessed based on EM analysis, MyD88 forms curved short fragments), which might make it more vulnerable to protease‐based inhibition.

### 2.5 Structural Justification of The Anti‐Inflammatory Property of LasB

**Figure 5:**
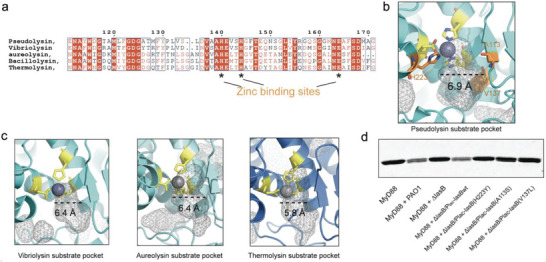
Structural justification of the anti‐inflammatory property of LasB. a) Sequence alignment of different M4 peptidases showing high level of conservation in zinc binding sites. b) Binding pockets of M4 peptidases from different bacterial species showing the conserved zinc binding residues in yellow and size of the neck of the binding pocket. Comparison of these binding pockets with LasB showed that LasB possessed a larger pocket at 6.9 Å. Vibriolysin (3NQX), Aureolysin (1BQB), Thermolysin (1TLX) c) Binding pockets of LasB. Residues in yellow are conserved zinc binding residues. Residues in orange make up part of the LasB binding pocket. LasB (1U4G) d) In vitro assay of MyD88 digestion showing abrogated activity of mutant LasB. H223 is a conserved residue that binds to substrates, while A113 and V137 are found in the binding pocket of LasB. Mutating either one of them caused the loss of LasB ability to digest MyD88.

Zinc metallo‐proteases, such as LasB, are common in many bacterial species.^[^ ^37^ ^]^ However, among the twelve bacterial secretions we examined, several bacteria expressing LasB homologs did not exhibit immune‐suppressive activity. We conducted a comprehensive structural analysis of related LasB homologs to identify the biochemical features contributing to its anti‐inflammatory activity. Zinc metallo‐proteases possess a conserved HEXXH motif that is important for its catalytic function. The two histidine residues and a downstream glutamate residue bind to a zinc ion, which is important for substrate binding and hydrolysis.^[^ ^38^ ^]^ Sequence alignment of the amino acid sequences around the substrate binding pocket of several M4 family peptidases (**Figure** **5**a) revealed high sequence similarities. This made it difficult to identify the reason LasB was able to degrade Death Domain adaptors in host cells. Thanks to the past accumulation of structural data, we compared the 3D structure of the active site pocket of these LasB homologs. As illustrated in Figure 5b,c, LasB had the widest substrate binding groove among all homologs we have compared, with a neck distance of 6.9 Å. A wider opening would endow this Zinc metallo‐protease with the ability to accommodate rigid loop or turning points of alpha helices, which are the potential cleavage sites in Death Domains. To validate our hypothesis, we introduced mild point mutations near LasB catalytic pocket that slightly narrowed the neck distance of the substrate binding groove. All three individual mutants (H223Y, A113S, and V137L) narrowed the groove by 0.6–0.8 Å, and this perturbation was sufficient to significantly abolish LasB's enzymatic activity to digest MyD88 DD (Figure 5d).

### 2.6 MyD88 DD was Cleaved by LasB at A24L25 Site, Which is Needed to Stabilize the Interface of the Active Signaling Oligomer

**Figure 6:**
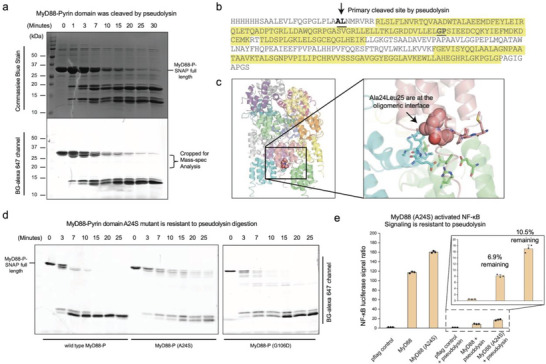
MyD88 DD was cleaved by LasB at A24L25 site. a) Coomassie blue and BG‐alexa647 fluorescent scans showing the gradual appearance of several new bands of MyD88 DD after LasB digestion. New bands appeared after 3–7 min digestions were pooled for Mass‐spec analysis. MyD88 DD was tagged by SNAP domain to labelling purposes. b) Mass spectrometry of the cropped protein bands indicated that boundary of remaining MyD88 fragments. Highlighted amino acid sequences were the peptide fragments identified by mass spectrometry. Except the first stretch of α‐helix, bulk of the Death domain was resistant to LasB treatment. A24L25 stood out as the likely cleavage site. c) Cartoon illustration of the location of A24L25 motif in MyD88 DD. Although it locates at the N‐terminal end of Death domain, this motif is important for stabilizing the oligomer interface. (PDB:3MOP) was used for illustration purposes. d) Side‐by‐side comparison of LasB digestion of wild type MyD88 DD, and its A24S mutant and a parallel G106D mutant. Only A24S showed significant protection against LasB digestion. e) HEK293T NF‐κB luciferase assay of MyD88 or MyD88(A24S) when subjected to LasB treatment. When electroporated the purified LasB directly into the model cell line using Neon transfection machine, MyD88 (A24S) showed relatively higher remaining signal. Mean ± standard error is from three independent experiments.

To identify the potential cleavage site on MyD88 Death domain, we performed analytical mass‐spectrometry to identify the peptide fragments after LasB digestion. We started with a time dependent digestion profiling of MyD88 (**Figure** **6**a), followed by cropping the faster migrating bands that newly appeared during the digestion process. These peptide fragments were pieced together later, as shown in Figure 6b. A24L25, which also fit the general prediction of LasB substrate preference, started to emerge as the most likely cleavage site. Interestingly, this motif is located at a kink in the first α‐helix of the MyD88 DD, making it more accessible by external factors, as shown in Figure 6c. At the same time, this potentially exposed motif is important for stabilizing a multimeric MyD88 complex, which is essential for TLR signaling.

We then proceeded to validate whether preventing LasB digestion at this specific structural motif could potentially resist LasB's effect. A single point mutation, Ala24Ser (LasB prefers hydrophobic or Gly rich motif), was introduced into MyD88 construct, and we tested whether this MyD88 mutant is resistant to *P. aeruginosa*‐induced immunosuppression in vitro and in vivo. In a recombinant assay (Figure 6d), MyD88 DD (A24S) significantly resisted LasB digestion, when compared to wild type MyD88‐DD. A parallel control mutant (G106D) does not confer MyD88 DD with such protection. In a cellular luciferase assay, MyD88 wild type and A24S mutant were overexpressed in HEK239T cells, when purified ≈40–50 nM LasB was electroporated into the cells, the MyD88 (A24S) resisted the immune‐suppression better than the wild type construct (Figure 6e). Interesting, we had observed consistently higher luciferase signal from MyD88 (A24S) at resting state, probably due to its resistance to other potential negative regulatory factors. Nevertheless, it is reasonable to propose that LasB from *P. aeruginosa* cleaves MyD88 at this position and subsequently suppresses the TLRs signaling pathway for its benefit.

### 2.7 LasB was Expressed and Secreted during Opportunistic Intracellular Infection of *P. Aeruginosa*

**Figure 7:**
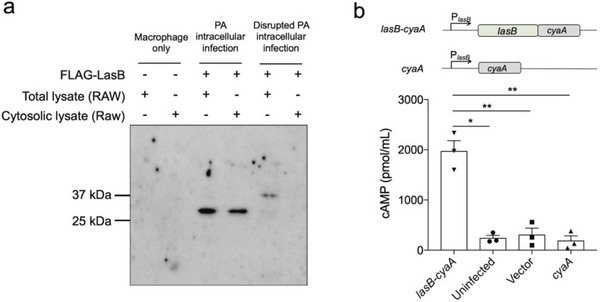
LasB is secreted and active during opportunistic intracellular infection of *P. aeruginosa*. a) Western Blot detecting anti‐Flag showed intracellular *P. aeruginosa* PAO1 Δ*lasB*/p*_lac_*‐*lasB*‐Flag actively expressing Flag‐tagged LasB in RAW264.7 macrophage cells. LasB was present in cytoplasmic fractions and total protein (cytoplasmic and secreted fractions). This indicated that *P. aeruginosa* expresses LasB intracellularly. But after the addition of erythromycin (disrupted PA intracellular infection group), LasB was not detected in either total protein or cytoplasmic fractions. b) RAW264.7 macrophages were infected with *P. aeruginosa* PAO1 harboring the indicated plasmids at MOI 50, and the cAMP level of cells was determined. Mean ± standard error is from three independent experiments. *0.01<*p*<0.05, **p<0.01, One‐way ANOVA.

Since we have shown that LasB is expressed during intracellular infection of RAW264.7 macrophage cells, we next tracked the localization of LasB inside macrophage cells using LasB Flag‐tagged at the C‐terminal. RAW264.7 macrophage cells were infected with *P. aeruginosa* containing Flag‐tagged LasB as above described. Immunoblot for anti‐Flag indicated that the LasB was actively secreted within the host cell cytoplasm (**Figure** **7**a). The secretion of LasB was further confirmed by adenylate cyclase (Cya) reporter assay.^[^ ^39^ ^]^ The Cya_2‐400_ reporter domain was fused to the C‐terminus of the full‐length LasB protein. Genes encoding the LasB fusion protein and Cya_2‐400_ domain were inserted into the pHERD20T plasmid, and transcription of the genes encoding these proteins was driven by the *lasB* promoter. We found that infection with *P. aeruginosa* producing the LasB‐Cya fusion resulted in a dramatic increase in RAW 264.7 macrophages intracellular cAMP levels. By contrast, infection with *P. aeruginosa* that produces Cya alone did not affect the cAMP levels (Figure 7b).

### 2.8 LasB Degrades MyD88 in Macrophage and Reduces the Level of TNF‐α

**Figure 8:**
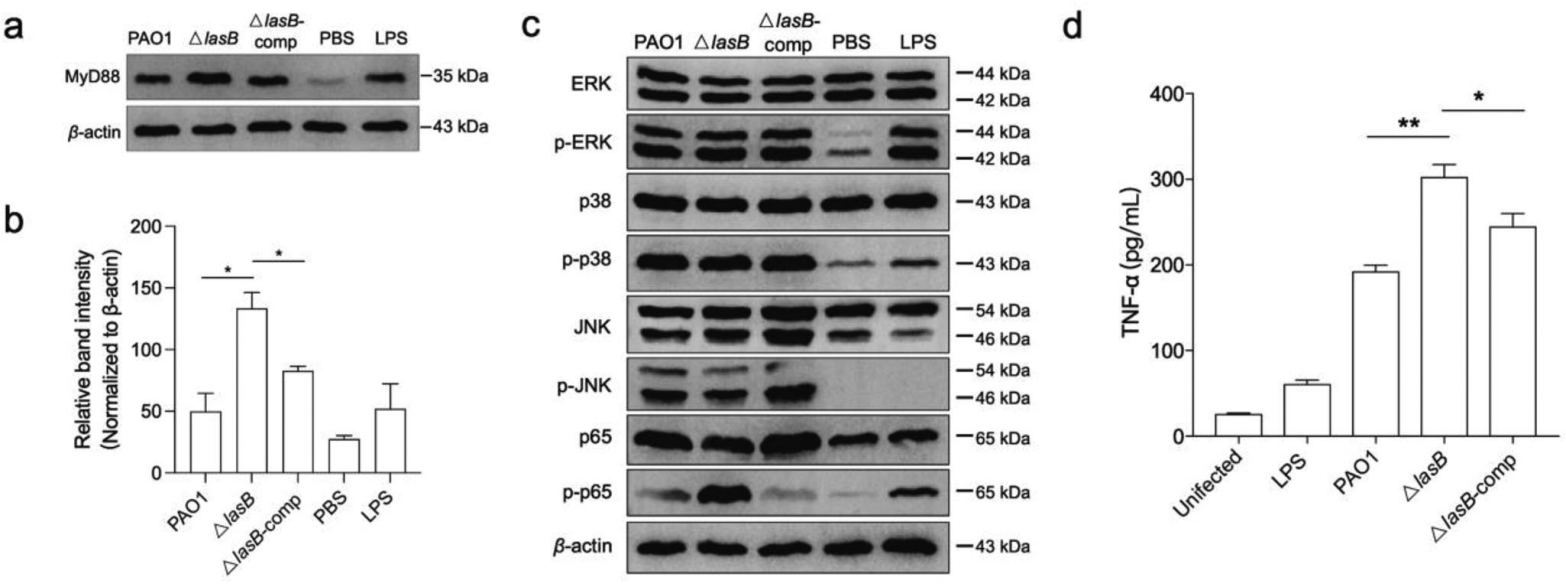
LasB degrades MyD88 in macrophage and reduces the level of TNF‐α. a) Western blot detecting endogenous MyD88 in RAW264.7 macrophages during the infection of PAO1, △*las*B and complement strain. b) Signal intensity of bands was measured by Image J software and each value was normalized to β‐actin signal intensity. Mean ± standard error is from two independent experiments. c) The protein levels of ERK, phospho‐(p‐)ERK, p38, p‐p38, JNK, p‐JNK, p65, and p‐p65were measured by Western blot analysis of the total‐protein extracts from RAW 264.7 cells, where *β*‐actin served as a loading control. d) TNF‐α release patterns in macrophages stimulated with the PAO1 strain and its mutants. Untreated and LPS treated macrophages were included as negative and positive controls. Mean ± standard error is from three independent experiments. *p<0.05, **p<0.01, ***p<0.001, One‐way ANOVA.

As an extracellular pathogen, *P. aeruginosa* is likely to initiate innate immune responses once internalized by the host immune‐surveillance cells. However, the above results suggest the secreted LasB by intracellular *P. aeruginosa* might quench the immune signal propagation to increase the chances of pathogen survival. To test the physiological effects of LasB on the immune response of the host, RAW264.7 cells were infected with PAO1, △*lasB* and the *lasB* complement strain, and the intracellular level of MyD88 was analyzed by western‐blotting. Compared to PAO1 and complement strain, we found that the intracellular MyD88 level was increased when infected with △*lasB* strain (**Figure** **8**a,b; Figure S5, Supporting Information). MyD88 is proximal to the TLR complex, the downstream signaling was also examined. Western blot data indicated that PAO1 attenuated the phosphorylation of the p65 subunit of NF‐κB and has no effects on the phosphorylation of ERK, JNK, p38 when compared to Δ*lasB* mutant (Figure 8c; Figure S6, Supporting Information). We further determined the production of TNF‐α and revealed that the level of TNF‐α was significantly increased in △*lasB* strain in comparison to PAO1 and complement strain (Figure 8d).

## 3 Discussion and Conclusion

*P. aeruginosa* causes human infections in the lungs, eyes, ears, bones, joints, heart, skin, and soft tissues and remains a principal cause of hospital‐acquired infections and ventilator‐associated pneumonia.^[^ ^40^ ^]^ In addition, the recognition of emerging antimicrobial resistance in this organism, including the global identification of circulating MDR and XDR strains, has placed this organism in the “critical” category of the published “dirty dozen” WHO list of organisms where novel treatment and intervention strategies are urgently required. There were a paucity of studies reporting how bacteria could suppress cellular innate immune responses.^[^ ^41^ ^]^ Bacteria could either subvert host innate immune responses inside host cells or secrete toxins to disrupt signaling cascades and silence downstream responses. Personnic and colleagues have showed that intracellular pathogen *Legionella pneumophila* is able to form virulent persisters within amoebae and activated macrophages, which produce distinct proteomes and require an active Lqs quorum‐sensing system.^[^ ^42^ ^]^ Recent evidence showed that extracellular pathogens such as *Staphylococcus aureus* and *P. aeruginosa* are also able to form persisters within infected host cells, which might constitute reservoirs for relapsing infections.^[^ ^43^ ^]^ To understand the rapid transition of bacterial physiology after entering host immune cells, we have developed the pulsed‐SILAC proteomics approach for systematically identifying the “newly synthesized” proteins after *P. aeruginosa* was engulfed by macrophages. Our study revealed a complicated active proteome for *P. aeruginosa* intracellular survival, including the induction of a range of proteins involved in the quorum sensing mechanisms. It is well known that bacteria employ quorum sensing regulated products to fight against the host immune systems and facilitate biofilm formation and antibiotic resistance.^[^ ^44^ ^]^ However, quorum sensing is generally considered an extracellular mechanism employed by bacterial cells, and how its regulated products function inside host cells remains largely unknown. In our study here, we are the first to reveal the *P. aeruginosa* quorum sensing regulated product LasB's immunomodulatory role in MyD88 degradation, which confers a novel function for the virulence factor typically implicated in extracellular infections.

LasB secreted by *P. aeruginosa* was first identified in 1949 by BalÓ and Banga^[^ ^45^ ^]^ and was considered the classic example of bacterial elastase for several decades.^[^ ^34^ ^]^ LasB can cleave collagen allowed *P. aeruginosa* to disrupt tissue integrity, allowing bacterial invasion.^[^ ^46^ ^]^ There are quite a few studies indicating that LasB is involved in suppressing the host immune functions during *P. aeruginosa* invasion by hydrolysing the host immune component such as IgA, TNF‐α, CD4 etc.^[^ ^47^ ^]^ Here, we showed that bacterial proteases are capable of reversing the formation of the innate immune signalosomes by efficiently cleaving the Death Domains oligomers, and we expanded the substrate range of LasB. This provided an alternative mechanism to a previous report that only human CARD‐only proteins^[^ ^43a^ ^]^ and Pyrin‐only proteins (POP) could bind to the end surfaces of active signaling ASC complexes and restrict the activity of the apoptosome.^[^ ^48^ ^]^

Although previous studies of innate immune signalosome demonstrated that innate immune signalosomes are self‐catalyzing and are fairly resistant to extreme biochemical conditions,^[^ ^49^ ^]^ the mechanisms for the breakdown and negative regulation of these complexes are not well understood. Our reconstituted approach made it possible to study purified fractions of secretions from several pathogenic bacterial species. Similar approaches could be very useful in dissecting the molecular mechanisms by which other intracellular bacteria pathogens, including *Mycobacterium tuberculosis* and *Vibrio cholerae*, modulate host responses. We demonstrated that MyD88 in macrophages was inhibited by LasB. Compared to other inflammasomes (ASC,^[^ ^49^ ^]^ MAVS,^[^ ^50^ ^]^ RIP2^[^ ^51^ ^]^), which are more rigid and larger in size, Myddosome is intrinsically smaller and more flexible. This makes MyD88 an easier target for a protease‐based inhibitor. Hence, the inhibition of Myddosome could explain how bacteria like *P. aeruginosa*, could prevent effective immuneresponses from being elicited against microbial infections.

Our finding also highlighted the role of elastase‐like proteases, which could cleave innate immune adaptor signalosomes previously thought to be non‐degradable. Human cells also express many different proteases that can degrade extracellular matrix. Considering the similarities in substrate, these proteases may potentially play a negative regulatory role for innate immune responses. Further investigation on how proteases, both human and pathogenic in origin, could shed light on regulating human innate immune responses. Moreover, identifying the enzyme capable of cleaving and inhibiting innate immune adaptor signalosomes provided tantalizing possibilities for potential inhibitors of such signaling complexes. Based on our study, we propose that employment of quorum sensing inhibitors (QSI) to suppress *P. aeruginosa* virulence products such as elastase would enhance the host immune clearance against its infections, which is to a certain extent similar to the application of PD‐1/PD‐L1 checkpoint inhibitors in tumor immunotherapy. In summary, we demonstrated a new type of pathogen‐host interaction where degradation of adaptor protein death domains allows intracellular survival in macrophages and highlighted the potential application of quorum sensing inhibitors in treating persistent *P. aeruginosa* infections.

## 4 Experimental Section

### Bacterial Strains and Growth Conditions

*E. coli* DH5a strain was used for DNA manipulations. Lysogeny broth (Luria‐Bertani, LB) medium was used to cultivate *E. coli* strains. Batch cultivation of *P. aeruginosa* strains was carried out at 37 °C in LB or ABTGC (ABT minimal medium supplemented with 2 g L^−1^ glucose and 2 g L^−1^ casamino acids). The medium was supplemented with 100 µg mL^−1^ ampicillin (Amp), 15 µg mL^−1^ gentamicin (Gm), 15 µg mL^−1^ tetracycline (Tc) or 8 µg mL^−1^ chloramphenicol (Cm) for the plasmid maintenance in *E. coli*. 30 µg mL^−1^ Gm or 150 µg mL^−1^ carbenicillin (Carb) were used for marker selection in *P. aeruginosa*.

### Cell Culture Models

HEK293T and murine macrophage cell line RAW264.7 cells were cultured in DMEM (Gibco) supplemented with 15% (v/v) fetal bovine serum (Gibco) under 37 °C, 10% CO_2_ condition. RAW 264.7 cell line was generously provided by Dr. Ruedl Christiane. Cells were incubated in 25 cm^2^ cell culture flasks at a density of 5.0 × 10^5^ cells mL^−1^ at 37 °C, 5% CO_2_ and 99% humidity for 48 h.

### RAW264.7 Macrophages Infection Model

As previously described,^[^ ^52^ ^]^ RAW264.7 macrophages were cultured in 24‐well plates at a concentration of 5 × 10^5^ cells mL^−1^. The cells were then exposed to bacteria at a multiplicity of infection (MOI) of 100:1 and incubated for 1 h at 37 °C, 5% CO_2_, and 99% humidity. After washing the macrophages with phosphate‐buffered saline (PBS) three times, the remaining extracellular bacteria were eliminated using Gm (100 µg mL^−1^) and the macrophages were further incubated for 4 h. The infected macrophages were lysed with 500 µL ddH_2_O containing 0.1% Triton X‐100, and the cell lysates were collected for downstream analysis. Cytokine quantification was performed using ELISA with the cell lysates. Bacterial numbers were quantified by serially diluting the cell lysates and plating them on LB agar plates, followed by incubation at 37 °C for 16 h. The number of colonies was counted, and colony forming units (CFU)/mL was calculated. The experiments were performed in triplicate, and the results were presented as mean±s.d.

### Pulsed‐SILAC

The experiment was carried out twice as independent biological replicates (BR1 and BR2) (Qinglian Bio, China). PAO1 △*lysA* was cultured overnight at 37 °C with shaking at 200 rpm in ABTG medium supplemented with 0.25 mg mL^−1 13^C_6_^15^N_2_ L‐lysine (Lys8, Thermo Fisher Scientific, USA) and 1.92 mg mL^−1^ Amino acid Drop‐out Mix Minus Lysine without Yeast Nitrogen Base (United States Biological, USA). The ^13^C labeled *P. aeruginosa* cells were then incubated with macrophage cells for an hour at a multiplicity of infection of 100:1 to facilitate phagocytosis. After washing macrophage cells with PBS, any remaining extracellular bacteria were removed using the Gm protection assay. The macrophages were then incubated for 4 h, washed with PBS, and lysed with PBS containing 0.5% Triton X‐100. The cell lysates were centrifuged at 500 g for 5 min to remove cell debris, and the bacteria were collected by centrifugation at 5000 g for 5 min. Samples from the DMEM control group underwent similar collection and processing. The pellets were collected and stored in liquid nitrogen for downstream analysis.

### Protein Extraction, Digestion, and Desalination

Proteins were extracted from the sample using 8 M urea and 10% protease inhibitor. After centrifugation at 14100× g for 20 min, the supernatant was collected and protein concentration was determined using the Bradford method. The remaining sample was stored at −80 °C. A 100 µg aliquot of extracted proteins from each sample was reduced using 10 mM dithiothreitol (DTT) solution and incubated at 37 °C for 1 h. Then, 40 mM iodoacetamide (IAM) was added and kept at 25 °C for 45 min. The sample was diluted 4 times with 25 mM ammonium bicarbonate (ABC) buffer. Trypsin (trypsin: protein = 1:50) was added and incubated at 37 °C overnight. The digestion was stopped by adding 50 µL 0.1% FA. The C18 column was washed with 100 µL 100% ACN, and centrifuged at 1200 rpm for 3 min. The eluents of each sample were combined and lyophilized. The resulting product was stored at −80 °C until further use.

### LC‐MS/MS and Data Analysis

The peptides were fractionated using high‐pH HPLC to reduce sample complexity. The process included dissolving peptides in buffer A (2% acetonitrile, pH 9.5) and loading them onto an Xbridge C18 column. Elution was performed at a flow rate of 0.7 mL mi^−1^n with a 70‐minute gradient from 0 to 95% buffer B (98% acetonitrile, pH 9.5). The eluted peptides were combined into three fractions for further analysis. For MS analysis, the separated peptides were analyzed using an Orbitrap Fusion Lumos instrument with a Nanospray Flex (ESI) ion source. The instrument performed full scans ranging from m/z 300 to 1400 with a resolution of 120000 (at m/z 200). The top 25 precursors of the highest abundant in the full scan were selected and fragmented by higher energy collisional dissociation (HCD) and analyzed in MS/MS, where resolution was 15 000 (at m/z 200), the automatic gain control (AGC) target value was 2 × 10^4^, the maximum ion injection time was 30 ms, a normalized collision energy was set as 30%, an intensity threshold was 5 × 10^4^, and the dynamic exclusion parameter was 15 s. The raw data of MS detection was named as “raw”.

The fractions were analyzed individually against the *P. aeruginosa* PAO1 database using Proteome Discoverer 2.4 (Thermo Fisher Scientific, USA). The analysis parameters were set to trypsin digestion with a maximum of two missed cleavages, minimum and maximum peptide lengths of 6 and 144 amino acids, respectively. A precursor ion mass tolerance of 15 ppm and a product ion mass tolerance of 0.02 Da were used. Carbamidomethyl (57.021 Da) was specified in PD 2.4 as static modifications and isotope labeled l‐lysines were set as dynamic modifications. Oxidation modification (+15.995 Da) of Met residues and acetylation of the N‐terminus were specified in PD 2.4 as dynamic modifications. A target‐decoy‐based false discovery rate (FDR) for peptide and protein identification was set to 0.01.

### Extraction of Bacterial Secretion

Bacterial strains were grown at 37 °C, 200 rpm in 5 mL LB for 16 h. The bacteria cultures were centrifuged at 13,000 g for 5 min. The supernatants were filtered through a 0.2 µm‐filter (Nalgene, USA) and stored at −80 °C.

### Molecular Cloning

To obtain the recombinant proteins needed for the assay, the genes encoding Myd88 DD^20‐117^, IRAK4 DD^27‐106^ and ASC PYD^1‐91^ were amplified using PCR from a cDNA library and cloned into pET47‐SNAP to obtain pET47‐Myd88 DD^20‐117^‐SNAP, pET‐47‐IRAK4 DD^27‐106^‐SNAP and pET‐47‐ASC PYD^1‐91^‐SNAP. To generate the LasB mutants, site‐directed mutagenesis was performed using KAPA Hifi PCR kits (KAPA biosystems) and confirmed by sequence (Figure S7, Supporting Information). The resulting constructions were transformed into BL21(DE3). The proteins were expressed and purified from the bacteria using Ni‐NTA agarose beads. The death domain proteins purified from bacteria tend to be short filaments and refolding was required to obtain functional monomers. Therefore, ASC PYD^1‐91^‐SNAP and IRAK4 DD^27‐106^‐SNAP (henceforth referred to as ASC PYD‐SNAP and IRAK4 DD‐SNAP respectively) was denatured using guanidine and refolded using multi‐step dialysis.

### Protein Purification

Recombinant SNAP fusion proteins were expressed and purified using BL21(DE3). Bacterial expression plasmids were transformed into BL21(DE3) cells, which were then cultured in LB with appropriate antibiotics. After reaching an OD_600_ of 0.6, induction with 0.5 mM IPTG occurred at 18 °C for 16 h. Cell lysis was performed at 25000 PSI using a lysis buffer (20 mM Tris‐HCl pH 8.0, 300 mM NaCl, 20 mM imidazole, 10% Glycerol), followed by centrifugation at 20 000 g for 20 min. Soluble proteins were captured on Ni‐NTA agarose beads (Biobasic), washed, and eluted using an elution buffer (20 mM Tris‐HCl pH 8.0, 300 mM NaCl, 300 mM imidazole, 10% Glycerol).

*P. aeruginosa* proteins were initially fractionated using gel filtration with Enrich SEC 650 (Bio‐rad) in phosphate buffered saline (PBS) at pH 7.4 (containing 137 mM NaCl, 2.7 mM KCL, and 10 mM phosphate buffer). The active fractions were then dialyzed in pH 5.0 Buffer A (50 mM sodium acetate) and subjected to cation exchange chromatography using HiTrap Heparin HP (GE Healthcare Life Sciences) with gradient elution in pH 5.0 Buffer B (50 mM sodium acetate, 2 M NaCl).

### Protein Refolding

The SNAP fusion proteins were expressed in BL21(DE3) cells at 18 ˚C for 16 h after induction with 0.5 mM IPTG. They were then purified using Ni‐NTA affinity chromatography. Following purification, the SNAP fusion proteins were briefly denatured in 6 M guanidinium hydrochloride at 37 ˚C for 30 min. To refold IRAK4 DD SNAP, the denatured proteins underwent sequential dialysis steps, first in buffer 1 (1.5 M guanidine, 20 mM Tris‐HCl pH 8.0, 3 M NaCl, 0.5 mM EDTA, 10% Glycerol, and 50 mM β‐mercaptoethanol) for 30 min at room temperature, then in buffer 2 (20 mM Tris‐HCl pH 8.0, 1 M NaCl, 0.5 mM EDTA, 10% Glycerol, and 50 mM β‐mercaptoethanol) for 30 min, and finally in buffer 3 (20 mM Tris‐HCl pH 8.0, 0.5 M NaCl, 0.5 mM EDTA, 10% Glycerol, and 50 mM β‐mercaptoethanol). After dialysis, the proteins were quickly processed: they were centrifuged at 14000 g for 1 min and filtered through a 0.22 µµ filter (Merck‐millipore). The refolded proteins were then labeled with either AlexaFluor 647‐benzylguanine or AlexaFluor 488‐benzylguanine, following the manufacturer's instructions, and immediately used for oligomerization assays.

### Oligomerization Assays

Monomer and seed were differentially labeled with 5 µM BG conjugated dyes (either BG‐647 or BG‐488) (New England Biolabs) and quenched with 10 µM of BG (Sigma). Monomeric protein obtained from refolding was mixed with seed for 15 min at room temperature to induce oligomerization. After oligomerization was induced, native loading dye (30% glycerol, 0.25% bromophenol blue, 0.25% xylene cyanol FF) was added and loaded onto 3–12% gradient native PAGE (Invitrogen). The gel was analyzed on Typhoon FLA 7000 (GE Healthcare Life Sciences) using the fluorescent imaging function at 635 and 473 nm excitation wavelengths to detect BG‐647 and BG‐488 stained proteins respectively.

### Strains Construction

In frame deletion of *lasB* was performed by allelic exchange method. Briefly, the 5′‐ and 3′‐ flanking regions of *lasB* were respectively amplified using primers lasB‐1/lasB‐2, and lasB‐3 /lasB‐4 with Q5 High‐Fidelity DNA Polymerase (NEB) using PAO1 genomic DNA as template, and purified with GeneJET PCR Purification Kit (Thermo Fisher). Subsequently, these two flanking fragments were assembled with the *Bam*HI and *Hind*III digested suicide vector pK18‐Gm with Gibson Assembly (NEB) and transformed into *E. coli* DH5α competent cells by heat shock at 42 °C. Transformants were detected with PCR using primers pK18‐F/pK18‐R. Positive transformants were grown and then triparentally mated with PAO1 under the help of RK600. Triparental cultures were spread on ABTC medium supplemented with 60 µg/mL gentamycin at 37 °C for 2 days. Conjugators were picked and recombinants were selected on ABTC medium containing sucrose (5%). Mutations were confirmed by PCR and DNA sequencing. For complementation, the coding region of *lasB* gene was amplified using the primer pair lasB‐EcoR I/lasB‐BamH I. The PCR product was purified and assembled with the *Eco*R I and *Bam*H I digested vector pUCP22Not, and then transformed into *E. coli* DH5α competent cells. The resultant plasmid was finally introduced into the *lasB* deletion mutant by the triparental mating as described above and detected by primers M13‐F/M13‐R. The complemented strains were confirmed by DNA sequencing.

For the p*lasB*‐*lasB*‐*cya* and p*lasB*‐*cya* construction, fragments p*lasB*‐*lasB* and p*lasB* were amplified using primers lasB‐5/lasB‐6, and lasB‐5/lasB‐7. The DNA sequence of Cya_2‐400_ for p*lasB*‐*lasB* and p*lasB* were amplified by cya‐1/cya‐3 and cya‐2/cya‐3. All the fragments were purified and assembled with the *Afl* II and *Hind* III digested pHERD20T with Gibson Assembly (NEB) and transformed into *E. coli* DH5α competent cells. The resulting plasmid were sequence and transformed into PAO1 by electroporation.

### Protein Transfection and Luciferase Assay

HEK293T cells were initially plated in 24‐well plates (Corning) at a density of 1 × 10^5^ cells per well, 24 h before transfection. Plasmids were introduced into the cells using FuGENE HD (Promega) as per the manufacturer's instructions, and the cells were allowed to recover for 16 h. LasB protein transfection was conducted using the NEON transfection system (Thermo Fisher) following the manufacturer's recommendations. After an 8‐hour incubation, cells were harvested and lysed using 1x Passive lysis buffer (Promega). Cell lysates were then aliquoted into 96‐well white‐bottom plates from Greiner. Firefly luciferase reporter activity was measured by adding Luciferase Assay Reagent II (Promega) to each well and recording the readings with a Tecan plate reader. Following this, Stop and Glow reagent (Promega) was added to each well, and Renilla luciferase reporter activity was similarly recorded using the plate reader.

### Negative Stain Electron Microscopy

IRAK4 DD SNAP was induced to form filaments mixing Myd88 DD SNAP at 0.07 and 0.7 uM respectively for 30 mins. Prepared immune adaptor filaments were adsorbed to carbon‐coated 200 mesh copper grids (Electron Microscopy Sciences) and blotted with filter paper and negatively stained with 4 µl of 2% (w/v) uranyl acetate solution. Images were collected using a Tecnai T12 (FEI) transmission electron microscope operated at 120 kV using a 4k × 4k Eagle (FEI Company) CCD camera at a nominal magnification of 490000× and a defocus value of −1.7 nm. The calibrated pixel size was 2.6 Å at the specimen level.

### Cya Assay

The RAW264.7 macrophages were replated into 24‐well culture plates. Triplicate cultures were infected with bacteria at a multiplicity of infection (MOI) of 50:1. After 2 h infection, cells were washed with PBS three times and then lysed with 50 mM HCl/0.1% Triton X‐100. After boiling for 5 min, extracts were neutralized with 6 µL of 0.5 M NaOH. The lysate was spun down at 13000× g for 5 min to remove macrophages and bacterial debris, the supernatant was used to determine the cAMP levels by an ELISA kit (mlbio, China). Experiments were performed with three replicates, and the results were shown as the mean±s.d.

### Confocal Microscopy

Microscopy images with brightfield, SYTO‐62 (Ex: 649 nm/Em: 690 nm), GFP (Ex: 535 nm/Em: 595 nm) were captured by the LSM780 (Carl Zeiss, Germany) confocal laser scanning microscope (CLSM) with a 40× objective. IMARIS software (Bitplane AG, Switzerland) was used to process the images. Experiments were performed in triplicate, with representative images being shown in this study.

### Western Blot

Antibodies targeting phospho‐p65 (p‐p65), p65, p‐JNK, JNK, pERK, ERK, p‐p38, p38, β‐actin, MyD88, and Flag M2‐Peroxidase were sourced from Cell Signaling Technology Inc.. Protein transblotting was accomplished using Mini Trans‐Blot Cell (Biorad) onto PVDF membranes, followed by blocking with 10% (w/v) blocking grade powder (Biorad) in TBST buffer (50 mM Tris pH 7.4, 140 mM NaCl, 0.1% Tween 20) for 1 h at room temperature. Subsequently, membranes were probed with either Anti‐MyD88 (1:800 dilution) or Monoclonal Anti‐Flag M2‐Peroxidase (1:5000 dilution) in TBST at room temperature for 1 h. The blot was washed three times with TBST with each time 10 min. Afterward, membranes were probed with an anti‐mouse IgG secondary antibody conjugated to horseradish peroxidase (Abcam) for 1 h at room temperature, followed by another round of three TBST washes. To visualize the results, ECL Prime Western Blotting Detection Reagent from Advansta was applied to each membrane and left for 1 min. The blot was visualized with Chemi‐Doc (Biorad).

### Cytokine Quantification by ELISA

TNF‐α concentrations were assessed using the CUSABIO TNF‐α ELISA kit, meticulously following the manufacturer's instructions. In brief, TNF‐α capture antibodies (diluted at a 1:250 ratio in 1× coating buffer) were coated onto Nunc Maxisorp 96‐well plates (Thermo Scientifics) and incubate at 4 °C for 16 h. Subsequently, the plates were washed three times with wash buffer (0.05% (v/v) Tween‐20 in PBS), followed by a 1 h blocking step at room temperature using 1x ELISA/ELISPOT diluent. RAW264.7 cell lysates, each containing 25 µg of protein and diluted in PBS, were then introduced to the wells and incubated at room temperature for 2 h. After an additional three washes, TNF‐α detection antibodies (diluted 1:250 in 1× ELISA/ELISPOT diluent) were added to the wells, followed by a 1 h incubation at room temperature. Subsequent to the washes, the plates were incubated at room temperature for 30 min with diluted avidin‐HRP (1:250), and a final series of five washes ensued. The reaction was initiated by adding 100 µL of tetramethylbenzidine substrate solution to each well, and absorbance at 370 nm was monitored every 5 min using a Tecan SPARK microplate reader until stable readings were stabilized.

### Statistical Analysis

The data were analyzed by two‐tailed Student's t‐test, one‐way ANOVA, or two‐way ANOVA with multiple comparisons. The data expressed as the mean ± SEM. GraphPad Prism software was used for statistical analysis unless otherwise indicated. Statistically significant differences were indicated as *p < 0.05, **p < 0.01, and ***p < 0.001.

## Conflict of Interest

The authors declare no conflict of interest.

## Author Contributions

X.D., Z‐Z.B., and S‐L.C. contributed equally to this work. X.D., Z.Z.B., and S.L.C. designed methods and experiments, carried out the laboratory experiments, analyzed the data, interpreted the results and wrote the manuscript. Z.L., K.H.C.C., R.Y., Y.P. performed the biochemical investigations. Z.J.S., B.J., M.M., S.S., S.H.C., F.G., advised and performed the in vivo investigations. Q.H., Y.L. and B. W. proposed the central hypothesis and wrote the manuscript. All authors have contributed to, seen and approved the manuscript.

## Supporting information

Supporting Information

Supplemental Table 1

## Data Availability

The data that support the findings of this study are available in the supplementary material of this article.
