# Supplementary material for: A Bacterial Quorum Sensing Regulated Protease Inhibits Host Immune Responses by Cleaving Death Domains of Innate Immune Adaptors
Source: Adv Sci (Weinh). 2023 Oct 23;10(34):2304891. doi: 10.1002/advs.202304891 (PMC10700182; doi:10.1002/advs.202304891)
Supplement: Supplementary file 1 — Supporting Information [file ADVS-10-2304891-s001.pdf]

ADVANCED SCIENCE
Open Access

Supporting Information

for *Adv. Sci.*, DOI 10.1002/advs.202304891

A Bacterial Quorum Sensing Regulated Protease Inhibits Host Immune Responses by Cleaving Death Domains of Innate Immune Adaptors

Xiangke Duan, Zhao Zhi Boo, Song Lin Chua, Kelvin Han Chung Chong, Ziqi Long, Renliang Yang, Yachun Zhou, Baptiste Janela, Sanjay Haresh Chotirmall, Florent Ginhoux, Qinghua Hu, Bin Wu* and Liang Yang**

Supporting Information

A bacterial quorum sensing regulated protease inhibits host immune responses by cleaving Death Domains of innate immune adaptors

Xiangke Duan[1,4,9]†, Zhao Zhi Boo[2,3]†, Song Lin Chua[5]†, Kelvin Han Chung Chong[2,3], Ziqi Long[2,3], Renliang Yang[2,3], Yachun Zhou[1,4], Baptiste Janela[6,7], Sanjay Haresh Chotirmall[6], Florent Ginhoux[8], Qinghua Hu[9]*, Bin Wu[2,3]*, Liang Yang[1,4]*

[1]Shenzhen Third People's Hospital, The Second Affiliated Hospital of Southern University of Science and Technology, National Clinical Research Center for Infectious Disease, Shenzhen 518112, China

[2]School of Biological Sciences, Nanyang Technological University, Singapore 637551, Singapore

[3]NTU Institute of Structural Biology, Nanyang Technological University, Singapore 636921, Singapore

[4]School of Medicine, Southern University of Science and Technology, Shenzhen 518055, Guangdong, P. R. China

[5]Department of Applied Biology and Chemical Technology, The Hong Kong Polytechnic University, Hong Kong SAR, China

[6]Lee Kong Chian School of Medicine, Nanyang Technological University, Singapore 639798, Singapore

[7]Skin Research Institute of Singapore, Singapore 308232, Singapore

[8]Singapore Immunology Network [1], Agency for Science, Technology and Research (A*STAR), 8A Biomedical Grove, Immunos, Singapore 138648, Republic of Singapore

[9]Shenzhen Center for Disease Control and Prevention, Shenzhen 518055, China

† Those authors contributed equally.

* Correspondence: huqinghua03@163.com, wubin@ntu.edu.sg, yangl@sustech.edu.cn

Supplementary Figures

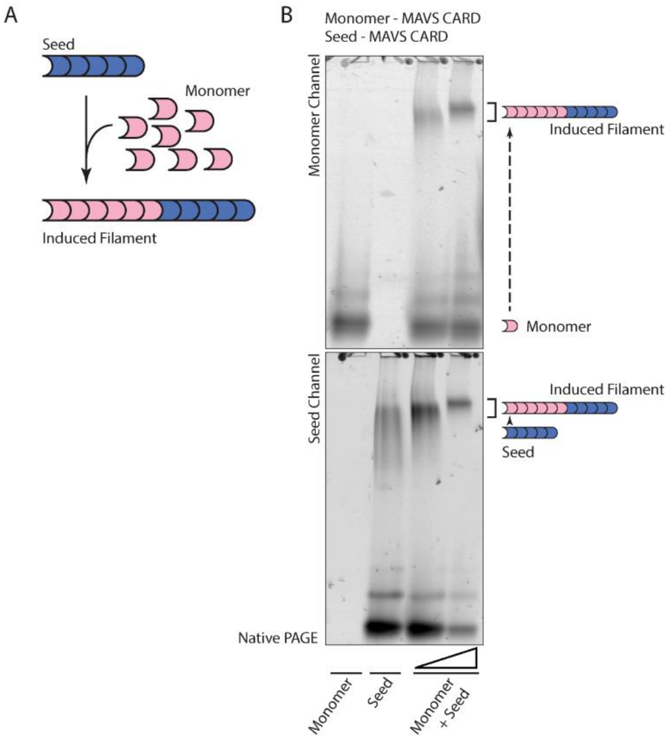

Figure S1. Schematic diagram of oligomerization assay. The oligomerization assay is derived from the previous publications,[2] and the was referred by other groups using indirect methods before.[3] On the left handside, using a MAVS oligomerization as an example. The SNAP tag linked seed oligomer and the monomeric fraction were separated labelled by two different fluorophores (BG-647 and BG-488). Visualized on two distinct fluorescent channels, the migration pattern of both seeds and monomers were monitored separately and their co-migration could be used to confirm induced oligomerization, hence a signal of activation.

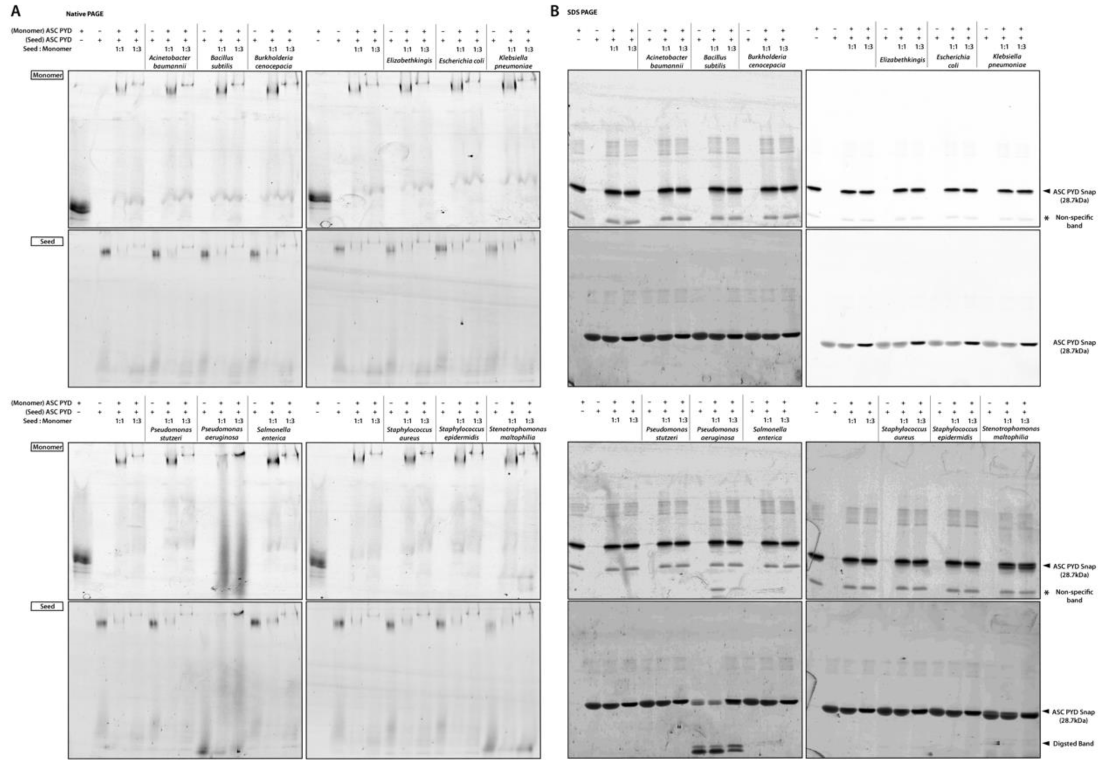

Figure S2. Raw images of secretomes analysis of 12 potentially pathogenic bacteria on the innate immune inhibition of MyD88 activation.

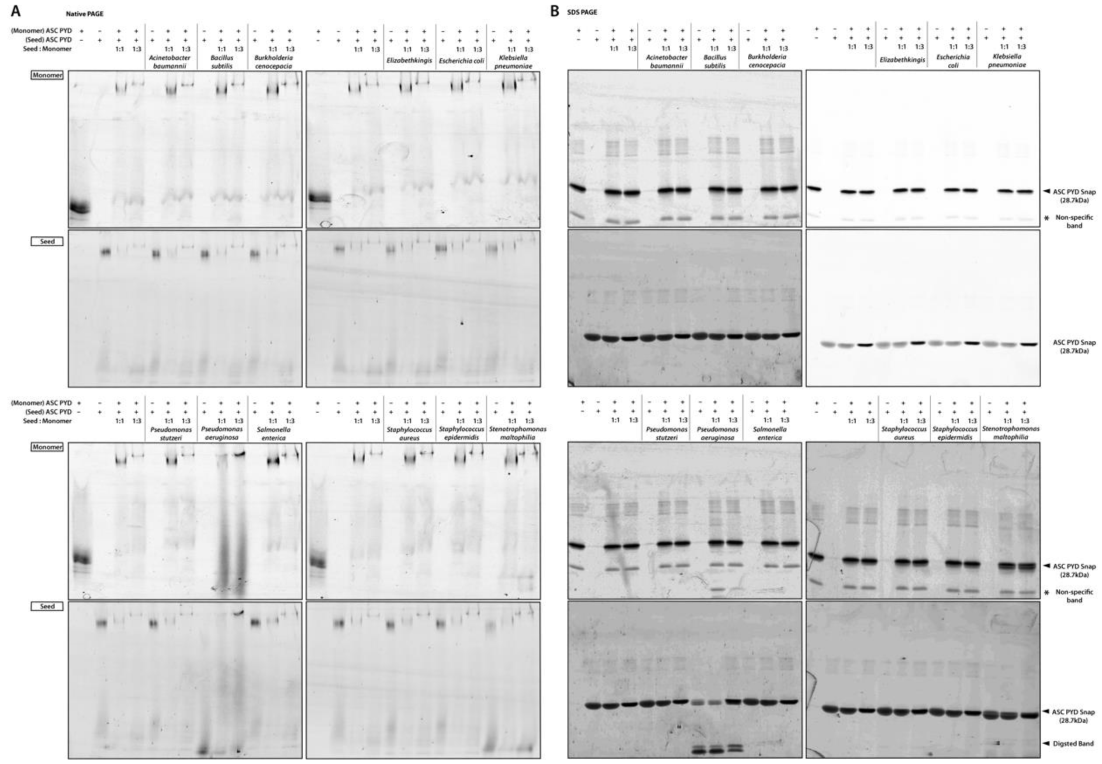

Figure S3. Raw images of secretomes analysis of 12 potentially pathogenic bacteria on the innate immune inhibition of ASC activation.

Nat_OligAss_IRAK4_2[Alexa Fluor 488] contrast

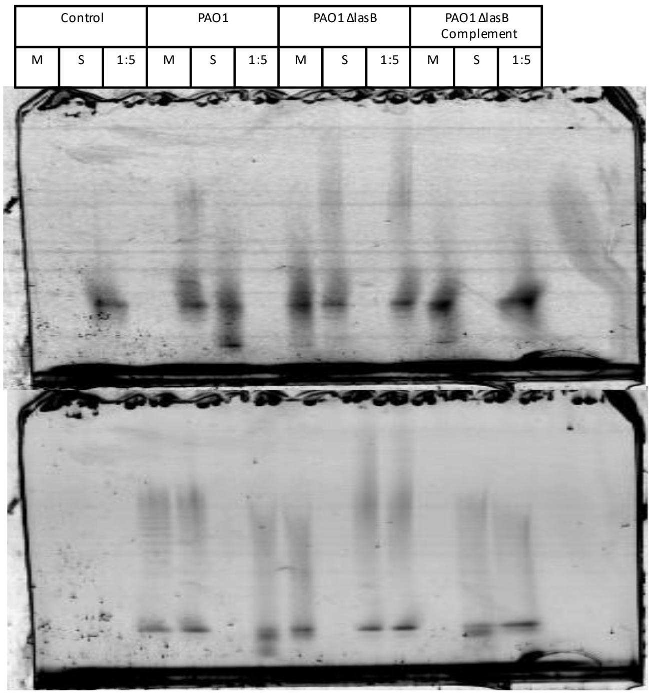

Figure S4. Raw images of secretomes analysis of PAO1, Δ*lasB* and complement strains on the innate immune inhibition of MyD88 activation.

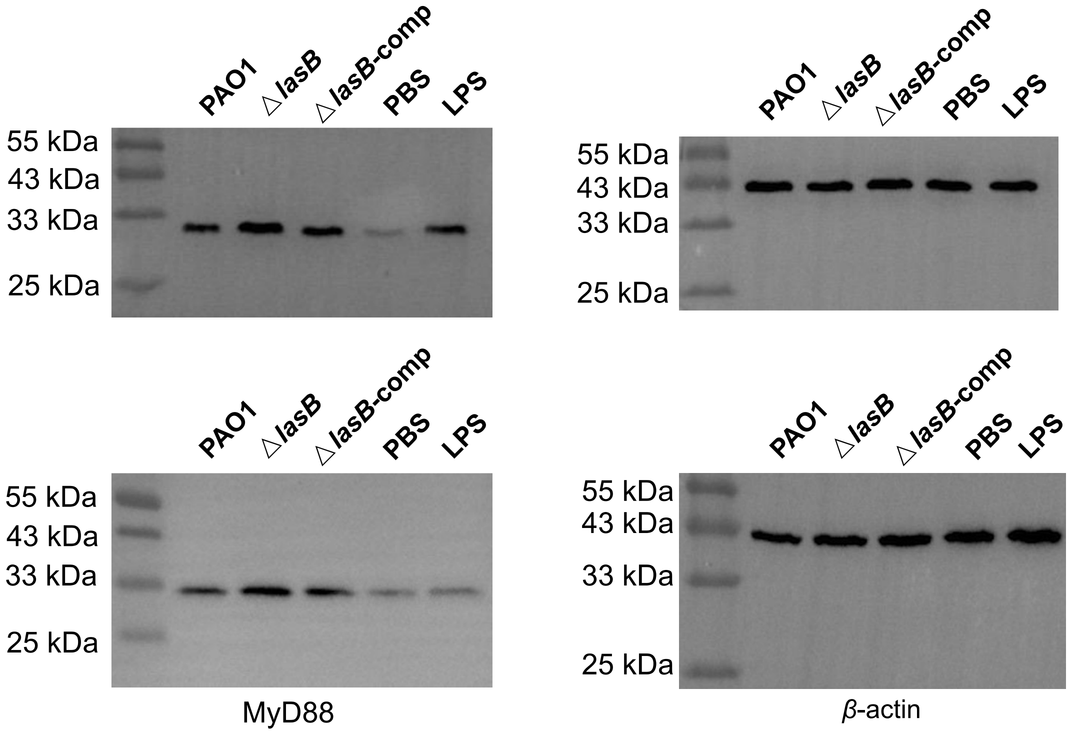

Figure S5. Raw images of western blot detecting endogenous MyD88 in RAW264.7 macrophages during the infection of PAO1, △*lasB* and complement strain.

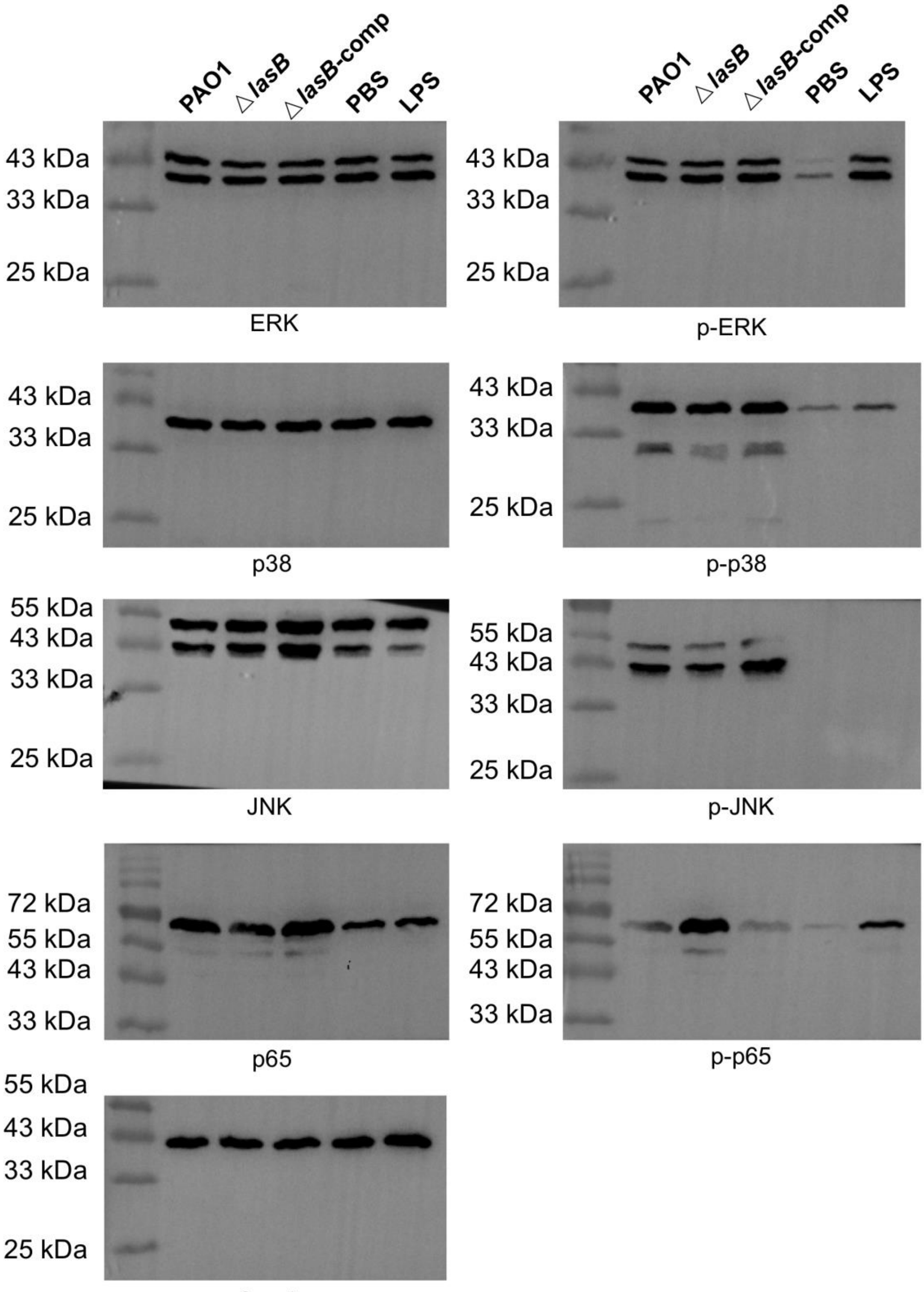

Figure S6. Raw images of the protein levels of MyD88, ERK, phospho-(p-)ERK, p38, p-p38, JNK, p-JNK, p65 and p-p65were measured by Western blot analysis of the total-protein extracts from RAW 264.7 cells, where *β*-actin served as a loading control.

pUCP_pseudolysin_H223Y

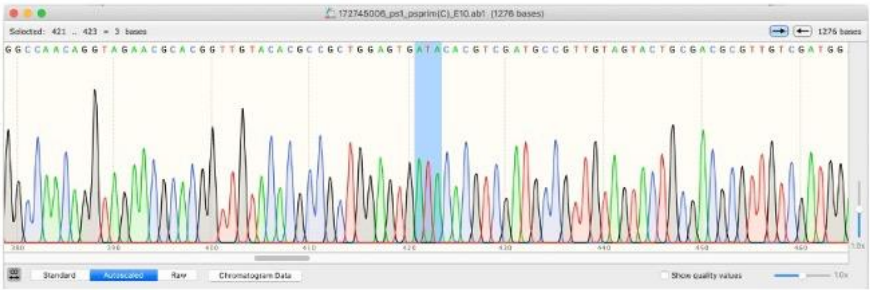

pUCP_pseudolysin_V137L

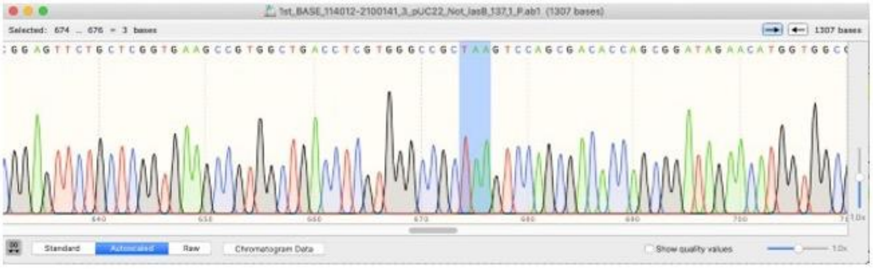

pUCP_pseudolysin_A113S

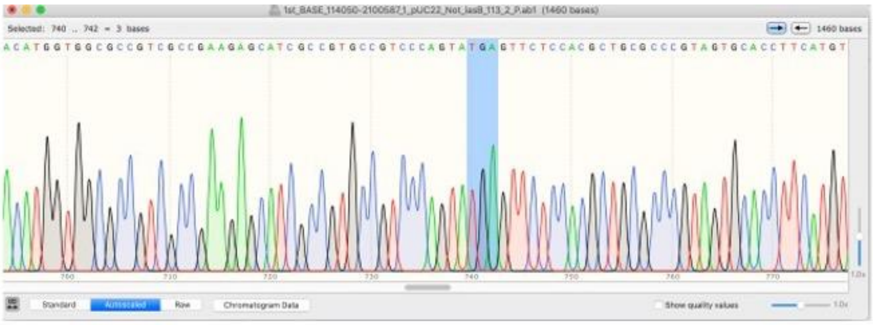

Figure S7. The sequence of pseudolysin point mutation.

Supplementary Table

Supplementary Table 2. Bacterial strains, plasmids and primers used in this study.

Strain	Characteristic	Source or reference
***P. aeruginosa* strains**		
PAO1	Prototypic non-mucoid wild-type strain	[4]
PAO1Δ*lysA*	L-lysine synthesis defective lysA mutant in PAO1	[5]
PAO1Δ*lasB*	Elastase defective *lasB* mutant in PAO1	This study
PAO1Δ*lasB*/p_{lac}-*lasB*	Gm^r; Δ*lasB* containing the p_{lac}-*lasB* complementation plasmid	This study
PAO1Δ*lasB*/p_{lac}-*lasB*$_{A113S}$	Gm^r; Δ*lasB* containing the p_{lac}-*lasB* plasmid with Residue 113 substituted from A to S	This study
PAO1Δ*lasB*/p_{lac}-*lasB*$_{H223Y}$	Gm^r; Δ*lasB* containing the p_{lac}-*lasB* complementation plasmid with Residue 223 substituted from H to Y	This study

PAO1Δ*lasB*/p_{lac}-*lasB*$_{V137L}$	Gm^r; Δ*lasB* containing the p_{lac}-*lasB* complementation plasmid with Residue 137 substituted from V to L	This study
PAO1-*gfp*	Gm^r; PAO1 containing the p_{lac} -*gfp*	[5]
PAO1-*lasB*-*gfp* (ASV)	Gm^r; PAO1 containing the *lasB*-*gfp* (ASV) translational fusion	[5]
E. coli		
DH5α	F^-, ø80d*lacZ*ΔM15, Δ(*lacZYA*-*argF*) U169, *deo*R, *recA*1, *endA*1, *hsdR*17(rK^-, mK^+), *phoA*, *supE*44, λ–, *thi*-1, *gyrA*96, *relA*1	Laboratory collection
DH5α /p_{lac}-*lasB*	Gm^r; Δ*lasB* containing the p_{lac}-*lasB* complementation plasmid	This study
DH5α /p_{lac}-*lasB*^{A113S}	Gm^r; Δ*lasB* containing the p_{lac}-*lasB* plasmid with Residue 113 substituted from A to S	This study
DH5α /p_{lac}-*lasB*^{H223Y}	Gm^r; Δ*lasB* containing the p_{lac}-*lasB* complementation plasmid with Residue 223 substituted from H to Y	This study
DH5α p_{lac}-*lasB*^{V137L}	Gm^r; Δ*lasB* containing the p_{lac}-*lasB* complementation plasmid with Residue 137 substituted from V to L	This study
**Other bacterial species**		
Acinetobacter baumanni		Laboratory collection
Bacillus subtilis		Laboratory collection
Burkholderia cenocepacia		Laboratory collection
Elizabethkingis meningtoseptica		Laboratory collection
Escherichia coli		Laboratory collection
Klebsiella pneumoniae		Laboratory collection
Pseudomonas syringae		Laboratory collection
Salmonella enterica		Laboratory collection
Staphylococcus aureus		Laboratory collection
Staphylococcus epidermis		Laboratory collection
Stenotrophomonas maltophilia		Laboratory collection
**Plasmids**		
pK18	Small mobilizable vector, Gm^R, sucrose sensitive (sacB)	[6]

pUCP22Not	*E. coli-Pseudomonas* shuttle vector, Amp^R Gm^R	[7]
pHERD20T	*E. coli-Pseudomonas* shuttle vector, Amp^R	[8]
**Primers (5'-3')**		
lasB-1	agctcggtacccgggTCGACCAGTTGAACCAGGAC	
lasB-2	GACCACCGAGCTTACAACGCCATCTTGTTCAGTTCTCCTG	
lasB-3	CAGGAGAACTGAACAAGATGGCGTTGTAAGCTCGGTGGTC	
lasB-4	cgacggccagtgccaGCCCTGGGATTCGATGAAAC	
lasB-5	ATGCAGCGGAAAGTATACCTTAAGGGCCAGGAAAGCGTGCAACTGATG	
lasB-6	TCCCCGGGTACCGAGCTCGAATTCCAACGCGCTCGGGCAGGTCACGCC	
lasB-7	AACCAGCCTGATGCGATTGCTGCATCTTGTTCAGTTCTCCTGGTTTTT	
pK18-F	TGCTTCCGGCTCGTATGTTG	
pK18-R	GCGAAAGGGGGATGTGCTG	
lasB-EcoR I	atgaccatgattacgCCAGGAGAACTGAACAAGAT	
lasB-BamH I	aggtcgactctagagGACCACCGAGCTTACAACGC	
cya-1	GGCGTGACCTGCCCGAGCGCGTTGGAATTCGAGCTCGGTACCCGGGGATCCggtggaggcggttcaggcggaggtggctctggcggtggcggatcgCAGCAATCGCATCAGGCTGGTTAC	
cya-2	AAAAACCAGGAGAACTGAACAAGATGCAGCAATCGCATCAGGCTGGTT	
cya-3	AAACGACGGCCAGTGCCAAGCTTttaCTGGCGTTCCACTGCGCCCAGC	

References

[1] C. J. Murray, K. S. Ikuta, F. Sharara, L. Swetschinski, G. R. Aguilar, A. Gray, C. Han, C. Bisignano, P. Rao, E. Wool, *The Lancet* **2022**, *399* (10325), 629.

[2] a) B. Wu, A. Peisley, D. Tetrault, Z. Li, E. H. Egelman, K. E. Magor, T. Walz, P. A. Penczek, S. Hur, *Molecular cell* **2014**, *55* (4), 511; b) B. Song, Y. Chen, X. Liu, F. Yuan, E. Y. J. Tan, Y. Lei, N. Song, Y. Han, B. D. Pascal, P. R. Griffin, *Immunity* **2021**, *54* (10), 2218.

[3] M. C. Moncrieffe, D. Bollschweiler, B. Li, P. A. Penczek, L. Hopkins, C. E. Bryant, D. Klenerman, N. J. Gay, *Structure* **2020**, *28* (3), 281.

[4] B. W. Holloway, A. F. Morgan, *Annual review of microbiology* **1986**, *40*, 79, https://doi.org/10.1146/annurev.mi.40.100186.000455.

[5] S. L. Chua, J. K. Yam, P. Hao, S. S. Adav, M. M. Salido, Y. Liu, M. Givskov, S. K. Sze, T. Tolker-Nielsen, L. Yang, *Nat Commun* **2016**, *7*, 10750, https://doi.org/10.1038/ncomms10750.

[6] A. Schäfer, A. Tauch, W. Jäger, J. Kalinowski, G. Thierbach, A. Pühler, *Gene* **1994**, *145* (1), 69.
[7] S. West, H. Schweizer, C. Dall, A. Sample, L. Runyen-Janecky, *Gene* **1994**, *148* (1), 81.
[8] D. Qiu, F. H. Damron, T. Mima, H. P. Schweizer, H. D. Yu, *Applied and environmental microbiology* **2008**, *74* (23), 7422.
